# Nature-Inspired Unconventional Approaches to Develop 3D Bioceramic Scaffolds with Enhanced Regenerative Ability

**DOI:** 10.3390/biomedicines9080916

**Published:** 2021-07-29

**Authors:** Andrea Ruffini, Monica Sandri, Massimiliano Dapporto, Elisabetta Campodoni, Anna Tampieri, Simone Sprio

**Affiliations:** Institute of Science and Technology for Ceramics, National Research Council, 48018 Faenza, Italy; andrea.ruffini@istec.cnr.it (A.R.); monica.sandri@istec.cnr.it (M.S.); massimiliano.dapporto@istec.cnr.it (M.D.); elisabetta.campodoni@istec.cnr.it (E.C.)

**Keywords:** 3D biomimetic scaffolds, ion-doped hydroxyapatite, self-hardening bone cements, bio-inspired mineralisation process, collagen, biomorphic transformation, bone regeneration, osteochondral regeneration, periodontal regeneration

## Abstract

Material science is a relevant discipline in support of regenerative medicine. Indeed, tissue regeneration requires the use of scaffolds able to guide and sustain the natural cell metabolism towards tissue regrowth. This need is particularly important in musculoskeletal regeneration, such as in the case of diseased bone or osteocartilaginous regions for which calcium phosphate-based scaffolds are considered as the golden solution. However, various technological barriers related to conventional ceramic processing have thus far hampered the achievement of biomimetic and bioactive scaffolds as effective solutions for still unmet clinical needs in orthopaedics. Driven by such highly impacting socioeconomic needs, new nature-inspired approaches promise to make a technological leap forward in the development of advanced biomaterials. The present review illustrates ion-doped apatites as biomimetic materials whose bioactivity resides in their unstable chemical composition and nanocrystallinity, both of which are, however, destroyed by the classical sintering treatment. In the following, recent nature-inspired methods preventing the use of high-temperature treatments, based on (i) chemically hardening bioceramics, (ii) biomineralisation process, and (iii) biomorphic transformations, are illustrated. These methods can generate products with advanced biofunctional properties, particularly biomorphic transformations represent an emerging approach that could pave the way to a technological leap forward in medicine and also in various other application fields.

## 1. Introduction: The Relevance of Biomimetism in Regenerative Materials

The treatment of bone defects is still a major problem in the orthopaedic field, and intensive research is dedicated to finding effective clinical solutions. Given the various drawbacks inherent in the use of autologous or heterologous bone grafts, which are still considered a gold standard of care for the treatment of bone defects, the main focus is to date on the development of synthetic scaffolds, capable to improve the osteogenesis and vascularisation in critical size bone defects.

Bone scaffolds are designed as devices able to be implanted into a bone defect and function as a supportive porous structure enabling cell attachment, new bone formation and proliferation, and progressive integration with the surrounding bone. These features are related to various physicochemical properties of the scaffold, effective at the multiscale, including bioactive chemical composition, grain size and topography, surface roughness and texturing, overall porosity, pore size, shape and interconnection, as well as adequate 3D architecture. Furthermore, to permit their complete replacement with the new bone, it is highly desired that the scaffold also exhibits bioresorption ability, compliant with the rate of new bone formation. All these characteristics should be met altogether in a bone scaffold. In fact, chemistry, texture, porous structure, and the overall 3D architecture can act as instructive signals for cells; however, only when in mutual synergy can they adequately promote the various biologic phenomena yielding new bone formation and maturation. On the one hand, the chemical composition and the surface features help to promote osteogenesis and new bone apposition, but only the presence of an interconnected porosity can permit extensive scaffold colonisation and integration with the new bone. Additionally, the chemical composition strongly affects also the bioresorption ability, but this feature is greatly enhanced by the presence of a nanosize texture and, again, by a cell-conducive, interconnected porosity that increases the specific surface area. On the other hand, the inverse relationship between porosity and mechanical performance urges scientists to develop new strategies to match such antithetic requirements so as to enable the use of the scaffold in effective clinical procedures and to comply with mechanical forces acting in vivo, particularly relevant when it comes to regenerate load-bearing bone segments such as the extremities.

Within such a scenario, among biomaterial scientists, there is increasing awareness of the importance to develop new concepts for bone scaffold manufacturing, biomimetic under various viewpoints, in the attempt to overcome the existing drawbacks and limitations in the biologic and mechanical performance [1,2]. The achievement of this target is made difficult by the great compositional and multiscale structural complexity of bone tissue, not possible to reproduce as a whole with conventional technological approaches. Indeed, bone tissue is composed of ~70 wt% of calcium phosphate (CaP) nanocrystals with an apatitic structure that is heterogeneously nucleated on a 3D collagenous matrix and includes various bioactive ions such as Mg2+ partially replacing Ca^2+^ or CO_3_^2−^ partially replacing PO_4_^3−^ (the so-called B carbonation). The disordered nature of this apatitic phase facilitates dynamic biologic processes regulating the bone metabolism, thus making such a biologic mineral phase a sort of ‘*living inorganic crystal*’. The biologic activity of bone is also supported by its unique 3D architecture which, owing to a hierarchical organisation developed from the molecular to the macroscopic scale, provides an outstanding mechanical ability, in turn, enabling self-repair and self-adapting mechanisms.

The classical approaches for 3D ceramic development, based on the sequence of powder processing–3D forming–sintering, cannot give material-retaining bioactive composition and hierarchical architecture typical of the natural bone tissue. Therefore, considering such technological barriers, and seeking effective, radically new strategies for the realisation of inorganic devices with biomimetic chemistry and 3D structure, material scientists are increasingly looking to nature, biologic phenomena, and living beings, many of which show outstanding mechanical performance related to their hierarchical structural organisation [3,4,5,6].

The present review is focused on recent strategies employed in the design and development of biomimetic materials for bone regeneration. Chemical biomimesis is well illustrated by apatitic nanoparticles, endowed with ion doping that enhances the bioactivity and antibacterial properties, and in the following, recent approaches will be discussed that aim to achieve such nanocrystalline phases with their unique biologic properties in the form of nanostructured 3D scaffolds, which is the real challenge in biomaterials science. In this respect, various nature-inspired approaches could help to develop new bioceramics, representing potential next-generation devices capable to overcome existing limitations and making a substantial leap forward in orthopaedic regenerative applications.

## 2. Bioactive Ion-Doped Nanohydroxyapatites

Hydroxyapatite (Ca_10_(PO_4_)_6_(OH)_2_, HA) is the most widespread material among the calcium phosphates used as bioceramics because its composition is very close to the mineral component of human bone and tooth enamel. Many previous papers reported the realisation of HA scaffolds as bone substitutes (i.e., maxillofacial, dental, and bone substitutes) or in the form of nanoparticles (HA-NPs).

The intrinsic bioactivity, biodegradability, and biocompatibility of HA-NPs make them an appealing option as bone filler, or as carrier or delivery agent for genes, proteins, or drugs. In addition, the usefulness of making HA-NPs fluorescent or intrinsically magnetic opens up several opportunities in imaging or treatment (e.g., tumour ablation). Relevant aspects to be considered when developing HA-NPs for biological applications are their major physicochemical characteristics such as particles size, morphology, crystallinity, dosage, surface area, aggregation, and impact on the cell fate and behaviour in vivo; furthermore, the response of HA-NPs is cell dependent [7,8].

The stoichiometric HA phase contains calcium (Ca) and phosphorus (P) at a molar ratio of 1.67 (Ca/P). Conversely, the biologic apatite forming the mammal bones is usually nonstoichiometric and Ca defective, with Ca/P ratios varying from 1.5 to 1.67, affecting the biological properties of HA and its biosolubility due to the hydrolysis rate increasing at lower Ca/P ratios. The crystal lattice of HA is characterised by a loose-packed structure permitting to host various ionic species, substituting calcium, phosphate, and hydroxyl ions. PO_4_ groups represent the base structure of the HA lattice, whereas two types of Ca atoms (I and II) are more liable and can be easily replaced by foreign monovalent, divalent, or trivalent ions without altering their P6_3_/m crystal symmetry space group. [9] A mechanism through which the accommodation of foreign atomic types is achieved entails the formation of nonstoichiometric Schottky defects due to combined vacancies on oppositely charged lattice sites. The accumulation of defects generates lattice distortions and describes why the crystallinity of ion-doped HA is usually, yet not always, poorer than that of its pure analogue.

**Figure 1:**
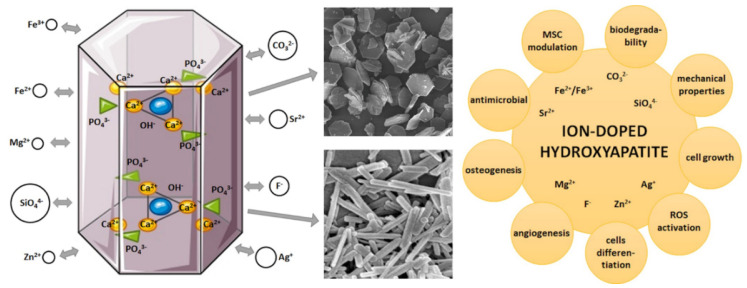
Biomimetic hydroxyapatite: (**left**): crystal structure and multiple ion doping determining different particle nanostructure; (**right**): scheme of the multifunctional applications of apatite nanoparticles.

Various methods have actually been adopted to enhance the bioactivity response of HA-NPs that include the polarisation of the surface area, or doping HA with cations or anions in replacement of PO_4_^3−^ or OH^−^ [10]. Doping with ions into HA has received much interest as this technique can simulate natural apatite, including trace ions such as Zn^2+^, Sr^2+^, Mg^2+^, Fe^2+^, Na^+^, Cl^−^, F^−^, K^+^, SiO_4_^4−^, CO_3_^2−^ ions [11]. These trace ions play a crucial function in the biochemical processes connected with bone metabolism and may affect the crystallisation, mechanical properties, degradation, and biological activity of apatite (Figure 1).

In order to obtain biomimetic materials mimicking the natural apatite in composition, structure, and function, ion-doped hydroxyapatite has been extensively investigated. Ion substitutions trigger an alteration of the lattice geometry, as induced by the different ionic radius and valence of the doping element. When a replacement with bivalent ions occurs, there is no charge imbalance within the apatite lattice, whereas, in the case of replacement with monovalent ions, the related charge unbalance yields the formation of extra vacancies, to reestablish the neutrality. It is also possible that multiple cationic and anionic doping coexist, thus influencing the crystal size and atomic arrangement in a nontrivial fashion but, to a certain extent, without destroying the typical hexagonal geometry of the apatite phase. The accommodation of cations either in the substitution of Ca(I) or Ca(II) within the lattice is strongly associated with their ionic radius: a cation with a larger radius than Ca^2+^ tends often to occupy site (II) because it is larger in volume than (I).

In what follows, several applications and properties of ion-doped HA are reported. The list of the most common doping ions is shown in Table 1.

Magnesium-doped HA (MgHA) enhances the solubility and biodegradability in physiological media and affects positively the biological effect by improving the osteointegration and osteoblasts activity, and accelerates bone ingrowth [12]. Mg doping enhances integrin-ligand binding and appears to protect the cells by oxidative stress [13]. MgHA facilitates new bone formation and apposition on bone implants, controlling the initial dissolution rate and addressing bone regeneration [14]. It was previously demonstrated that MgHA has significant antibacterial and larvicidal ability against various pathogens [15], particularly bacteria such as *Staphylococcus aureus*, *Pseudomonas aeruginosa*, and *Escherichia coli* [16].

The presence of Strontium ions incorporated in the hydroxyapatite lattice (SrHA) increases biocompatibility and bioactivity and promotes the osseointegration process. It was demonstrated that SrHA promotes osteoblasts differentiation and proliferation, enhancing the activity of alkaline phosphatase (ALP), as well as the production of collagen type I and osteocalcin [17]. Thanks to its ability to hinder osteoclasts’ activity, reducing their proliferation and thus enhancing the osteogenesis process, strontium-doped hydroxyapatite can be relevant in the treatment of osteoporotic patients in cases of tumour resection or trauma [18,19]. Many biological investigations were carried out on this doping ion, in particular by verifying its interaction with osteoblast-like cells and human osteoclasts to verify proliferation or inhibition, osteogenic differentiation, and angiogenic factor expression. It has been concluded that the bone cell behaviour is Sr^2+^-ion-dose dependent [20,21,22]. It was also shown that Sr doping can stabilise the HA crystal which affects, on the one hand, the mechanical stability of the new bone and, on the other hand, also increases the hardness of ceramic scaffold [23,24].

The doping of hydroxyapatite with Iron ions was carried out to achieve new magnetic materials for medicine. In a previous study, HA was doped with both Fe^2+^/Fe^3+^ ions, thus achieving an apatitic phase (FeHA) with intrinsic superparamagnetic ability while retaining also excellent biocompatibility, osteogenic ability, and bioresorbability, opening to new advanced applications in nanomedicine, such as magnetic cell targeting or hyperthermia therapy for cancer, where a significant reduction in tumour volume was observed after injection of FeHA and treatment inside the magnetic field [25,26]. After injection, magnetically labelled cells (e.g., mesenchymal stem cells) can be driven by static magnetic fields and localised to the target site where they can perform their specific role. This approach can be transferred to different cell types as an effective magnetic carrier of drugs, growth factors, miRNA, etc., offering great perspectives in nanomedicine [27].

Other cations frequently used as doping ions for hydroxyapatite are Zinc (ZnHA) and Silver ions (AgHA). Zinc revealed effects similar to strontium and often Sr^2+^ and Zn^2+^ ions are included in hydroxyapatite simultaneously to achieve synergistic effects [28]. Zinc ions promote the bone mineralisation processes by enhancing osteoblasts’ proliferation, cell growth, and differentiation while inhibiting the bone resorption by osteoclasts. A low dosage of zinc demonstrated effective bioactivity and antibacterial properties, as inhibitory action on the growth of bacteria and fungi; ZnHA can be used as coating of dental implants, because of being able to promote tooth remineralisation and reduce bacterial adherence and tartar development [29,30]. Concerning the biological response to AgHA, many studies aimed to verify the more appropriate amount of added silver, in order to reach a favourable compromise between the anti-infective ability and cytotoxicity [31,32].

Carbonate ion (CO_3_^2−^) is the major substituent in biological hydroxyapatites and is present in a content of 4–8 wt%. It is accepted that carbonate ions mainly replace phosphate species (B type) in biological apatite, while in synthetic apatite, it can replace both hydroxyl groups (A type) and phosphate groups [33]. B-type carbonated apatites are characterised by a mechanical reinforcement of the bone and incremented solubility [10]. Carbonate hydroxyapatite (CHA) has been shown to increase the bioactivity and degree of osteoconductivity, due to its greater solubility than noncarbonated HA [34]. Increased collagen production by osteoblast cells was evidenced on the CHA, ascribed to increased extracellular calcium concentrations that resulted greatly higher in cell culture medium containing CHA, which promoted the secretion of type I collagen [35].

Fluoride (F^−^) is an essential anion for the natural apatite composing the dental enamel, and also is utilised for the treatment of initial caries lesions to prevent tooth decay [36,37]. Its presence in HA can increase osteoblast proliferation and differentiation and simultaneously exhibit antibacterial properties. The incorporation of fluorine ions within the apatite lattice easily occurs, even at room temperature, substituting for hydroxyl groups in the so-called anion channel [38]. It has been reported that fluoride ion stimulates extracellular matrix formation in vitro and enhance bone union, promoting osteoblasts activities, cell proliferation, and differentiation. Fluoride is considered an important therapeutic agent in osteoporosis treatment and can enhance proliferation and alkaline phosphatase activity.

The incorporation of Silicate ions (SiO_4_^4−^) into the phosphate unit network of the HA stimulates bone formation and resorption processes, which are relevant to both tissue regeneration and bone growth. In addition, silicon is essential for other biological soft tissue functionality, such as cartilage growth [39]. It was reported that silicon stimulates and improves osteoblast-like cell activity in vitro, enhances bioactivity, and induces a higher dissolution rate in vivo [40].

**Table 1:** Most common doping ions and their influence on biological properties of hydroxyapatite. The ionic radii (Å) of stoichiometric hydroxyapatite ions are Ca^2+^ 0.99; PO_4_^3−^ 2.38; OH^−^ 1.40.

**Doped Cations**	**Ionic Radius (Å)**	**Main Properties**	**Reference**
Mg^2+^	0.69	enhanced biodegradability improved biocompatibility osteogenic antibacterial	[12,13,14,15,16]
Sr^2+^	1.13	improved biocompatibility osteogenic inhibit osteoclast activity improve mechanical properties	[17,18,19,20,21,22,23,24]
Fe^2+^/Fe^3+^	0.84/0.66	drug targeting bioseparation hyperthermia therapy growth of osteoblast	[25,26,27,41,42]
Zn^2+^	0.74	osteogenic promotes osteoblast proliferation cell growth and differentiation antibacterial	[28,29,30]
Ag^+^	1.28	antibacterial	[31,32]
**Doped Anions**	**Ionic Radius (Å)**	**Main Properties**	**Reference**
CO_3_^2−^	1.78	enhanced solubility enhanced biodegradability improved biocompatibility osteoconductivity increased collagen production	[10,33,34,43]
SiO_4_^4−^	2.40	biomineralisation osteogenic increased bioactivity growth of osteoblast enhanced cell proliferation more efficient differentiation	[39,40,44,45]
F^−^	1.19	biomineralisation osteogenic less soluble in acidic solutions influences cell behaviour and responsiveness antibacterial	[27,28,29]

Hydroxyapatite, in its nanosize form, can be realised by a variety of techniques. The most exploited synthesis processes are carried out in wet conditions, such as precipitation, hydrothermal, sol–gel, and biomimetic deposition. It is necessary to carefully regulate the process conditions during the synthesis (pH, reaction time, temperature, concentration, type and state of precursor, etc.) to obtain the desired chemical composition and physical and morphological characteristics of the final hydroxyapatite. The synthesis of mono- or multiple-substituted hydroxyapatite is usually performed by following the typical processes applied for the undoped material. Table 2 briefly highlights the common approaches of synthesising HA, typical conditions, and characteristics of the final product.

The precipitation process is performed at atmospheric pressure, low temperature, and inside a reaction batch.

The form, size, and surface area of the realised HA using the precipitation procedure heavily depend on the reaction rate and temperature. Instead, the sol–gel process can create apatite crystals at lower temperatures as well as pressure due to the higher reactivity of the precursors. This approach also improves the chemical uniformity of the HA produced due to the fact that it includes the mixing of phosphorus and calcium in the atomic state. The main drawback of the sol–gel approach is the long time required to gel HA, although this problem can be resolved by executing the reaction at higher pressures and temperature levels. The primary difference between the precipitation and sol–gel approaches is that the first one includes precipitation of HA with the product remaining in the aqueous medium, whereas the latter includes gelation of the entire mixture. As for the biomimetic deposition approach, it has been shown that chemical precipitation of calcium nitrate and diammonium hydrogen phosphate salts, occurring in simulated body fluid (SBF) at 37 °C and pH 7.4, can create pure and homogeneous HA powders. In this procedure, the use of SBF can support the growth and also the generation of bone-like calcium apatite on submerged materials, at a physiological pH and temperature. The hydrothermal technique enables the development of well-crystallised HA powder. In this approach, the synthesis is performed by the availability of water at high temperatures and pressure. Heating the reactants in a closed reactor generates a pressurised system as the temperature increases, according to the water phase diagram.

The synthesis method based on neutralisation of alkaline Ca-containing suspensions allows the crystallinity and the ion content to be modulated by acting on the temperature and the ionic strength [46,47], thus enabling multiple doping. In this respect, it was found that multiple ion doping, better reproducing the complex composition of inorganic bone, enhances the cell behaviour. On the other hand, it was also found that multiple ion doping enhances antibacterial ability, as activated by surface charges and nanocrystallinity, as recently observed. This finding has great relevance for biomedical applications, opening the opportunity for new strategies that develop new materials with multifunctional biological abilities [48,49]. In particular, inherent antibacterial properties are very precious, in consideration of the ever-increasing bacterial resistance to antibiotics and the steady raise of nosocomial infections which are among major causes of therapeutic failure. Ion doping also permits the activation of specific biofunctionalities such as luminescence [50,51,52] or intrinsic magnetic properties [53,54]. In this latter case, the doping of HA with divalent and trivalent Fe ions (FeHA) could be modulated to yield specific positioning of the two ions in the HA lattice and within the hydrated external layer, in turn, activating dipolar interactions and interplay generating intrinsic magnetisation properties. Considering the very good biocompatibility, bioresorbability, and osteogenic ability of such a magnetic apatite, in contrast with cytotoxic magnetic metal oxides, such a finding may open the opportunity for numerous applications in nanomedicine such as smart drug delivery system, magnetically guided nanoparticles for cell and gene therapy, and also in regenerative medicine to advance the osteogenic character of endogenous cells, particularly suitable in the case of patients with compromised endogenous abilities such as the elderly [55,56].

Nanocrystalline, ion-doped apatites are of great interest for applications in medicine. However, in the form of free nanoparticles, they are not useful for bone regeneration as they would be if developed as 3D solid scaffolds. As anticipated in the previous paragraph, a major obstacle in this respect is represented by the chemical and thermal instability of nanocrystalline, ion-doped apatites: under the thermal sintering process required for the consolidation of ceramic scaffolds, apatitic nanophases lose their bioactivity and resorbability. The next sections will show some recent approaches to overcome such an impasse and develop a 3D scaffold retaining bioactive composition and nanostructure.

## 3. Chemically Consolidated Calcium Phosphates as 3D Injectable Scaffolds

Under certain clinical conditions, such as an age-related bone fracture (often related to osteoporosis), bone reconstruction entailing solid scaffolds might not be a practical approach. A negative aspect of existing orthopaedic devices is the need to obtain the implant in a specific shape and fit it in the bone gap. This can cause increases in bone loss, damages to the surrounding tissue, and extended surgical time. A basic requirement for tissue-engineered bone grafts is the ability to integrate itself with the host bone while supplying load-bearing ability and capacity to favour the bone remodelling process. Therefore, minimally invasive alternatives are sought, for instance, in maxillofacial treatments, or in osteoporotic vertebral fractures. These latter cases are the most common among the elderly, particularly due to the predominant trabecular structure of vertebral bones [57], more subjected to progressive thinning and microarchitectural alterations with respect to cortical bones. As an alternative to palliative pharmacological therapies or external immobilisation, for decades, vertebral fractures have been treated with injectable bone cements (IBCs) [58,59].

IBCs can be infused in the bone gap to create a bioactive scaffold and show specific self-setting capacity in physiological conditions without further processing, which enables their application in different fields. For instance, typical applications are surgical procedures that have been introduced to healing vertebral compression fractures, vertebroplasty (VP), and kyphoplasty (KP) [60].

IBCs may be classified into several species according to their chemistry: calcium phosphate cements (CPCs), calcium sulphate cements (CSCs), acrylic bone cements (ABCs), and composite cements (CICs) [61]. Between them, CPCs are the most attractive materials since they are biocompatible, osteoconductive, bioresorbable, and provide an intrinsic microporous structure for the transportation of nutrients and metabolic biological products [62]. CPCs offer an appealing outlook as an innovative class of injectable, applicable for the regeneration of bone defects with 3D complex geometry (e.g., femur head, tibial plateau, vertebral body, and maxilla) owing to the possibility of direct insertion in the osseous gap by mini-invasive surgical procedures.

Furthermore, a great advantage for the use of CPCs is that no sintering processes are required for their consolidation. This implies that the final hardened construct can retain the bioactive composition and nanostructured porous architecture, enhancing the bioactivity and biosolubility and particularly favouring the bioavailability of calcium and phosphate ions to cells.

In order to develop an ideal injectable bone scaffold for orthopaedic applications, where the bone regrowth is stimulated by biomimetic composition and by the ability of cells to penetrate inside the scaffold, a wide variety of properties should be satisfied, such as easiness to prepare, handling, injectability into the collapsed bone, clinically adequate working and setting time, and low curing temperature. All these features have great relevance for the handling and management of the CPC in effective clinical procedures. On the other hand, chemical composition, porosity extent, pore size distribution, and mechanical properties are, as a whole, very relevant for the biologic performance, because they are key aspects for cell adhesion, new bone apposition, osteoconduction, osteointegration, and bioresorption [63]. All these aspects are strictly interrelated so that the development of formulations converging all these relevant properties is a great technological challenge.

A multitude of formulations has currently been used to generate CPCs [62,64,65]. CPCs are obtained after mixing insoluble calcium phosphate with a liquid phase, which is usually water or an aqueous solution, although water-immiscible liquids have additionally been used to enhance handling and cement properties. Some available calcium phosphates used as insoluble powders in the CPC formulation, along with their composition, basic acronyms, and solubility information, are listed in Table 3 [9].

Upon mixing, the created paste can set after the bone cavity is filled, generating a calcium-phosphate-based viscous paste filling the bone void. The chemical composition of CPCs is close to that of the natural bone, which implies the release of Ca^2+^ and PO_4_^3−^ ions, promoting osteoconduction and osteogenesis. CPCs set as a result of dissolution and precipitation mechanism, while the physical entanglement of the precipitated crystals yields consolidation and hardening. Modifying powder-to-liquid proportions can lead to CPCs with a range of self-setting times. The setting time is defined as the time required for the cement hardening, starting from the mixing of the solid and liquid components. Reducing the powder-to-liquid ratio of CPCs raises the injectability while the setting time increases, also affecting the final mechanical properties [66].

To date, most of the CPCs so far developed are composed of precipitated hydroxyapatite (HA) or brushite (DCPD). This is expected, considering that HA is the most stable calcium phosphate at pH > 4.2, whereas brushite is the most stable at pH < 4.2.

As shown in the example of Table 4, CPCs can be obtained by various components in the solid phase (single or multiple), through different types of setting reactions (hydrolysis or acid base), setting mechanisms (dissolution–precipitation), and types of final product formed after injection.

The approach based on the hydrolysis of α-TCP is particularly interesting because it allows processing a single component that can be obtained by solid-state reactions, thus giving rise to pure apatitic bone cement. α-TCP is metastable at temperatures lower than ~1150 °C, and thus, βTCP phase can recrystallise during the cooling of α-TCP. Foreign ions such as Sr^2+^ and Si^4+^ can be introduced in the structure of α-TCP during its synthesis, thus affecting its reactivity with water and the hydrolysis kinetics, but also the formation of ion-doped apatite upon setting, potentially affecting the biologic and mechanical performance of the set cement [67,68].

**Figure 2:**
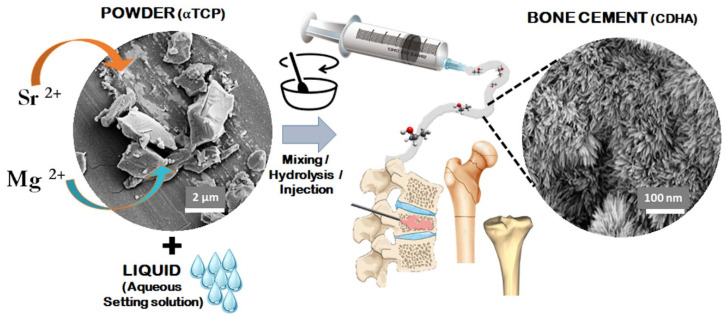
Scheme showing the mechanism of formation of apatitic bone cements and their applicability in bone regeneration.

When in contact with water, during the setting process, the recrystallisation process forms a network of intertwining elongated particles that can assume different shapes including needle-like morphology, favoured by small particle size, with higher surface area and supersaturation degree. On the contrary, a larger plate is favoured when the particles are larger. Additionally, the powder size influences the pore size in the final construct: basically, the smaller the particles of the solid component are, the smaller the pores in the set cement are [69] (Figure 2).

One of the most crucial issues of CPCs is the control of the setting responses after chemical reaction and rheological properties to reach appropriate injectability, setting time, and mechanical properties. The porosity and microstructure of CPCs can be altered by modifying the process conditions, such as the liquid-to-powder proportion and the size of the powder [70]. The presence of pores in the set cement is very relevant for its biological and mechanical performance. Indeed, the physical entanglement of the elongated particles forming CPCs also yields the formation of pores, with pore size, however, limited to micron and submicron size. Although porosity negatively affects the mechanical properties, a significant porosity extent is required to favour bone ingrowth, osteointegration, and bioresorption, some limitations occur when it comes to achieving macroscopic, cell-conducive pores [61,71]. Typically, the porosity of CPCs is in the range of 30–55% and is dependent on the liquid/powder ratio: higher ratios are related to an increase in porosity and vice versa [71]. Furthermore, the presence of a specific level of porosity makes this product also an excellent carrier for a controlled drug delivery system [72,73,74]. In this respect, the porosity of CPCs can be enhanced through the introduction of solid porogens, such as inorganic substances and polymers, or by the use of foaming agents [75]. A recent approach includes the enhancement of these characteristics by adding natural polymers or their derivatives water-soluble polymers [76] by mixing them with the powder in the early phase or directly adding them in solution into the paste. These ingredients, blended with the initial cement precursors, greatly influence the rheological properties and affect the setting time, viscosity, dispersion, plasticity, as well as compressive strength and toughness of the final device. For instance, the incorporation of natural polymers may help in regulating the viscosity and improve the injectability and cohesion, owing to their ability to cross-link under physiological conditions (T = 37 °C; pH ~ 7). In Table 5, some polymers most used for alternative formulations of CPCs and their evaluated properties are listed.

Furthermore, making use of biopolymeric ingredients can be also an effective method to improve the mechanical performance of CPCs [93]. Relying on the desired properties, the polymeric solution might be modified through variation of polymer/powder ratio, dimension of polymer beads, molecular weight, and polymer chain size [94,95]. The size and the extent of additives into the bulk calcium phosphate powder can influence the packing capability of CPCs. It has been demonstrated that the enhancement of fine fillers (about 1 μm in diametre) alters the packing capacity, minimises the water need, and raises injectability [61].

Within the numerous strategies proposed for the synthesis of CPCs, the incorporation of doping ions (Mg^2+^, CO_3_^2−^, SiO_4_^4−^, Sr^2+^, etc.) inside the structure of the starting powder through an optimised synthesis [96,97] is particularly interesting for many biological advantages, as described in the previous section. α-TCP, used as a calcium phosphate source for CPCs’ formulation, is suitable to be doped for instance with Sr or Si ions, due to its metastable crystal structure [67,68].

Owing to the great incidence of osteoporotic fractures, particular emphasis was recently dedicated to strontium ion as a tool to promote bone regeneration. The presence of strontium involves cell proliferation and differentiation into bone-forming osteoblasts and decreases the resorbing activity of mature osteoclasts [98]; this is crucial for the repair of the bone turnover balance, particularly when the cement is utilised in the treatment of osteoporotic bone fractures. Furthermore, the introduction of strontium in the crystal lattice is associated with an increased solubility of the cements, increasing the ions release, which has a positive impact on cell proliferation and also on the osteogenic process [99]. In this respect, a recent study highlighted the relevance to conjugate an Sr^2+^-doped apatitic cement with a natural polymer such as alginate to obtain enhanced injectability through a surgical cannula and very good osteogenic ability and osteointegrability [68] in a rabbit study. Despite several promising results that have been obtained thus far, CPCs are still lacking important features permitting their extensive use in orthopaedics [69]. Indeed, since mechanical properties and porosity are inversely related, it is still hard to obtain CPCs with wide open and interconnected porosity without penalising the mechanical performance. However, the possibility to obtain 3D bioactive scaffolds, owing to the low-temperature hardening of CPCs, still encourages material scientists to develop injectable, self-hardening materials, with the attempt to overcome the use of acrylic cements, which are still considered as the preferred choice, due to their low cost, easy applicability, and prompt recovery of the physical stability, but are affected by various drawbacks and, as bioinert compounds, are unable to yield effective bone regeneration [100,101,102].

## 4. Three-Dimensional (3D) Hybrid Scaffolds for Regeneration of Multifunctional Anatomical Tissues

The musculoskeletal tissues constitute a complex organ system including bones, ligaments, tendons, and muscles, altogether interacting in synergy to ensure stability, manipulation, mastication, and movement. Particularly, certain anatomical districts such as the joints and the periodontium can suffer from serious degenerative diseases that result in high suffering for patients and relevant socioeconomic impact. In this respect, osteoarthritis (OA) is considered one of the most common degenerative diseases affecting the joints and presently ranks fifth among all forms of disability worldwide, with approximately 50 million people living in Europe with OA-related disability globally, a number which is progressively increasing due to an ever-growing aged population [103,104,105]. Osteochondral tissues consist of a subchondral bone surmounted by a cartilaginous tissue (the articular cartilage), organised in a peculiar arch-like architecture, providing outstanding mechanical properties [106]. OA causes progressive sclerosis of articular cartilage and subchondral bone, causing cartilage damage and changes in the subchondral bone architecture and leading to mechanical instability of the joint and loss of its mechanical function.

None of the current pharmacological therapies available (e.g., anti-inflammatory, systemic), nor the use of injectable systems that mimic healthy synovial fluid or prostheses are satisfactory solutions for the definitive recovery of the affected part, with the exception of small, non-load-bearing osteochondral tissues [107,108]. Therefore, it is increasingly believed that new regenerative approaches should be developed to heal osteocartilaginous tissues and achieve effective functional recovery [109,110]. A relevant bottleneck in this respect is that current regenerative therapies mainly focus on articular cartilage, neglecting subchondral bone damage that is always present in advanced OA [111]. Therefore, scaffolds designed for joint regeneration should exhibit features reproducing the complex compositional and structural heterogeneity of the whole osteochondral unit. The articular cartilage and its supporting subchondral bone represent a functional unit which is of paramount relevance for the joint mechanical performance [106]. In particular, the cartilage layer has complex viscoelastic properties and a unique hierarchical structure with a smooth, lubricated surface accounting for low friction. This allows the transfer of multi-axial loads to the underlying subchondral bone, with the thin tidemark layer minimising the stiffness gradient between the rigid bone and the more pliable cartilage [112].

**Figure 3:**
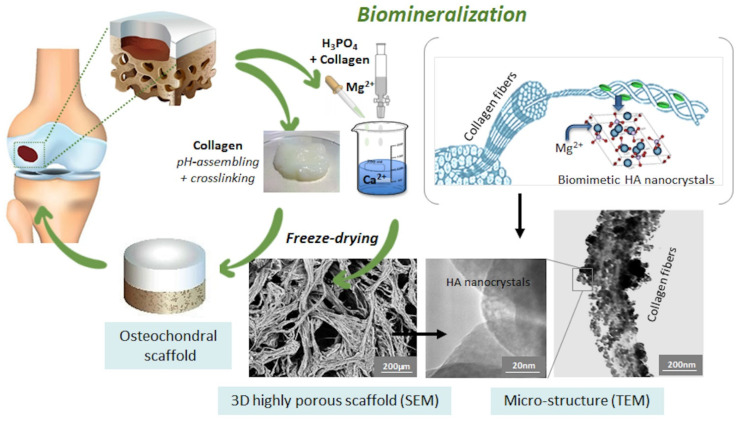
Scheme of the bioinspired mineralisation process to develop osteochondral scaffolds.

Attempts to generate osteochondral scaffolds often refer to the use of hydroxy-acid polymers conjugated with calcium phosphate particles under various approaches [113]. However, such methods were not able to reproduce the great compositional and structural complexity of osteocartilaginous tissues. In response to this challenge, a nature-inspired method based on biomineralisation was developed in recent years. Biomineralisation is a complex phenomenon by which natural organisms generate nanostructured hybrid constructs, as basic components of exo- and endoskeletons, dental tissues, and shells [114]. These constructs are characterised by inorganic nanocrystals grown on self-assembling bioorganic structures acting as templates and substrates guiding and controlling heterogeneous nucleation processes [115]. Particularly in bone tissue, the bioorganic matrix prevalently composed of collagen, besides providing charged functional groups able to link Ca^2+^ and other divalent ions, exerts a molecular and crystallographic control on the growing inorganic phase, limiting the extent of crystallisation and the crystal growth and rendering the inorganic phase itself biologically active [116]. Such a natural process was reproduced in the laboratory by exploiting the information stored at a molecular level within the collagen molecule. This information is relevant in guiding the supramolecular assembly of collagen into fibrils, during which the formation of thicker fibres are accompanied by heterogeneous nucleation of apatite nano-nuclei, activated at the carboxyl groups of collagens, where calcium ions present in the solution are initially bound. These competing mechanisms are activated by pH variation targeting the isoelectric point of collagen to induce its self-assembling and the pH range of stability of the apatitic phase (pH > 4.2) with respect to other calcium phosphates. If the process is carried out in the presence of additional ions such as Mg^2+^ and Sr^2+^, it results in the heterogeneous nucleation of ion-doped apatitic nanophases, particularly relevant for the enhanced interaction with cells (see also previous paragraphs). Through this process, mineralised hydrogels could be obtained and then transformed into hybrid fibrous constructs mimicking the composition and porous structure of the newly formed bone by a freeze-drying process (Figure 3).

The intimate conjugation of organic and inorganic components into such a hybrid composite yields unique properties based on their intrinsic characteristics. The first contributes to elastic properties and bending strength, while the second contributes to hardness and resistance to compression; these combined characteristics give these biomaterials unique properties that are difficult to realise by conventional synthetic materials. A relevant implication related to this bioinspired mineralisation process is the possibility to modulate the bioactivity, bioresorbability, hydrophilic properties, and mechanical performance by varying the composition and strength of the bioorganic template through cross-linking processes strengthening interfibrillar cohesion [117,118,119,120].

In addition, the mineralisation extent can be easily adjusted by varying the concentration of ions present in the reaction vessel. This opened the possibility to achieve scaffolds with graded mineralisation extent, thus enabling the preparation of monolithic devices recapitulating the compositional and structural complexity of multifunctional tissues such as the joint or the periodontium [121,122].

As a result of the very good mimicry of natural osteochondral tissues, graded hybrid scaffolds showed the ability to favour the formation of cartilage and bone tissue in the different histological layers and completely heal critical size osteochondral defects. In a sheep study, where critical size osteochondral defects were created, after six months from implantation on femoral condyles, the formation of new hyaline-like cartilage was observed, together with good integration of the scaffold with the cartilage, as well as an ordered columnar arrangement of the chondrocytes forming the subchondral trabecular bone. Compared to the control material, where no spontaneous healing was observed, the hybrid material showed complete resorption with the repair of the defect. This phenomenon was achieved without the aid of cell seeding or other growth factors, thus being among the first reports demonstrating that biomimetic compositional and structural features in a scaffold can effectively act as instructing signals for cells and yield effective tissue regeneration [123,124]. The scaffold was also tested in numerous preclinical and clinical trials, demonstrating excellent ability to direct cell phenotype and modulate osteogenic/chondrogenic cascade and regenerate critical size osteoarticular defects, also showing the successful remodelling of the original fibrocartilage tissue into hyaline cartilage [125,126,127].

Targeting dental regeneration, previous studies reported clinical trials where hybrid scaffolds were applied for regeneration of the alveolar bone. In one study involving 32 patients, the scaffold was implanted to achieve socket preservation and bone regeneration after tooth extraction, resulting in abundant bone regrowth [128]. In a different study, the hybrid scaffold was implanted into 15 patients for purpose of sinus augmentation, a preparatory process for the subsequent insertion of a dental implant. Additionally, in this case, abundant new bone formation, consistent with the implant resorption, was observed, resulting in substantial augmentation of the alveolar bone [129].

**Figure 4:**
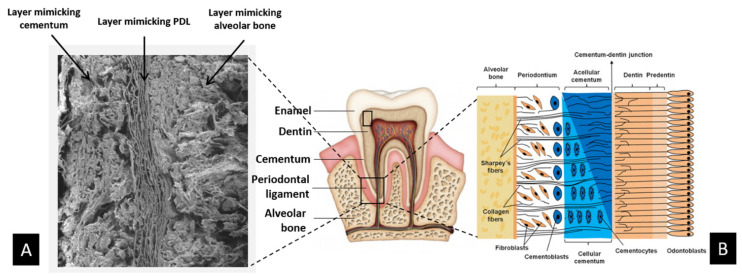
Scheme of multifunctional hybrid scaffold for periodontal regeneration: (**A**) SEM images showing at the microscale the multilayer scaffold made of three biomaterials mimetic of cementum, periodontal ligament, and alveolar bone; (**B**) details of the complex structure of natural periodontal tissue that inspired the fabrication of the three-layer biomimetic periodontal scaffold.

As in joint districts, dental tissues constitute an intricate complex of various mineralised and nonmineralised tissues, such as the alveolar bone, dentin and cementum, and the periodontal ligament. This tissue complex, named periodontium, acts as a functional unit constituting and supporting the tooth. Periodontal tissues can be damaged by chronic periodontal disease, such as periodontitis, which causes the loss of healthy teeth and is a cofactor involved in systemic diseases involving the cardiovascular and respiratory systems [130]. Biological mechanisms active in the formation of dental tissues are similar to those leading to the formation of bone and cartilage. Therefore, biomineralisation processes were investigated and conjugated with electrospinning techniques to achieve a hybrid construct with heterogeneous composition and architecture mimicking the complex formed by alveolar bone, periodontal ligament, and cementum [131] (Figure 4).

In this work, the scaffold was implemented with magnetic properties by using nanoparticles of hydroxyapatite partially substituted with Fe^2+^/Fe^3+^ ions (FeHA) embedded in the cementum layer. The use of magnetism in regenerative medicine is an emerging concept, considering the ability of static magnetic fields to stimulate cells and enhance osteogenic ability. In this respect, it was also shown that alternate or pulsed magnetic fields can help modulate the release of drugs or growth factors chemically linked to a scaffold, thus permitting more effective, personalised therapies [132,133,134]. In addition, owing to the high biocompatibility of FeHA, in comparison with iron oxides commonly used in medicine, FeHA can be easily internalised in human cells, which enables magnetic guiding, thus representing an opportunity for new cell-based therapies. In a different approach, magnetic hydroxyapatite was also introduced into hybrid scaffolds by the biomineralisation process. In this case, the magnetic phase was heterogeneously nucleated on self-assembling collagen fibres, thus obtaining a graded construct that mimics multifunctional osteocartilaginous regions but is also endowed with magnetisation properties, promoting cell proliferation and osteogenic differentiation [134].

Targeting a different dental district, characterised by very intricate architecture, scaffolds mimicking the dentine were recently obtained by using a gelatine–alginate blend, mineralised with Mg-doped HA by bioinspired mineralisation process [135]. Channelled and oriented porosity and channel size, obtained by controlled freeze drying and ionotropic gelation techniques, could be comparable to those of the natural dentin. These materials were tested in 3D cell culture with mesenchymal cells, stem cells from dental pulp, and odontoblast-like cells. Their biomimetic and hydrophilic composition facilitated cell adhesion and differentiation in osteoblasts, while porosity allowed for adequate long-term cell colonisation. The achievement of effective scaffolds enabling guided tissue regeneration in dentistry is highly desired today, in consideration of the difficult management of multiple cell lines acting in the oral environment. In this respect, the possibility to combine bioorganic and inorganic phases by nature-inspired assembling processes is promising in the generation of new smart bioactive devices showing anisotropic and graded physicochemical and mechanical features for the regeneration of complex multifunctional anatomical regions.

More generally speaking, biomineralisation processes reproduced in the laboratory offer a flexible tool to generate hybrid scaffolds with designed composition and multiscale structure, well reproducing the biologic microenvironment promoting the natural cell metabolism. The most serious drawback is given by the mechanical performance of hybrid fibrous scaffolds, insufficient to permit the use in load-bearing regions, even though, as reported above, the mechanical properties of hybrids can be modulated by various physical and chemical methods targeting interfibrillar cross-linking. A recent approach for obtaining reinforced hybrids is given by the possibility to conjugate collagen with other biopolymers, basing on their cytocompatibility and the possibility to be engineered into blends. Natural polymers such as nanocellulose, chitosan, alginate, fibroin (see in Table 6 [114]), are extremely available in nature and can be combined by activating specific mechanisms that govern relevant physical–chemical interactions [136,137,138]. A remarkable aspect is related to the possibility to use such natural polymers upon recovery from waste products [139,140]. This is today a critical problem, which is increasingly addressed by the overall community in order to find a way to reuse food waste in a circular economy approach. Hence, it can be envisaged that the incoming years will witness the surge of new technologies to process such critical but extremely useful raw materials for application in medicine and in many other applicative sectors.

## 5. Biomorphic Transformations: A Novel Approach to Generate Bone Scaffolds with Biomorphic, Hierarchical Architecture

The treatment of critical-size and nonunion defects in long, load-bearing bones of the limbs, originated by trauma, tumours, or other degenerative diseases, requires difficult and painful clinical procedures and, owing to numerous failures related to the insurgence of infections, pseudoarthrosis, and impaired bone regeneration, still represents a critical challenge in orthopaedics [156,157,158,159,160].

The regeneration of long bone segments is particularly critical as bone scaffolds targeting these districts must ensure superior biologic and mechanical performance. In fact, different from other bony regions, long bone segments are called to bear complex, ever-changing mechanical forces, and hence, the scaffold should be capable of yielding regeneration of bone tissue with its unique biomechanical ability. Therefore, a bone scaffold addressing long bone regeneration should be instructive in terms of osteoinductive chemistry, osteoconductive porous morphology, and effective mechanical performance while, at the same time, being also able to favour extensive vascularisation [161].

Seeking new bioactive devices for bone regeneration, previous sections highlighted interesting recent approaches where regenerative scaffolds were obtained in the form of 3D nanostructured porous constructs with osteoinductive chemical composition. Nevertheless, the need to conjugate open porosity with strong mechanical properties still prevented the use of hybrid or self-setting ceramic scaffold in critical-size load-bearing bone defects.

When using ceramic materials, the sintering process is the best-known method to achieve consolidated bodies. The sintering process is effective as it induces coalescence of the primary particles while reducing the intergranular void volume. However, if we focus on nanocrystalline, ion-doped apatites, reputed as golden biomaterials for bone regeneration, the sintering process yields its stabilisation into stoichiometric, microcrystalline hydroxyapatite, thus losing all its osteogenic and biosolubility properties, which are strictly related to the disordered, unstable nature of biological-like apatitic phases [162]. From a geometrical perspective, the pore coalescence related to the sintering process provokes the loss of the nanosize porosity, raises the specific surface area, and reduces the wettability. To enhance the mechanical strength, the overall porosity is often penalised, thus limiting the effectiveness in osteointegration and vascularisation, with the formation of necrotic zones [163]. As these drawbacks result from inherent features of ceramic materials, they are virtually unsolvable. Therefore, such an impasse impelled scientists to develop new approaches surpassing the traditional ceramic processing, through the development of radically new synthesis and consolidation procedures [1,5,164,165,166,167].

Seeking new biomimetic approaches, in recent years, scientists are rediscovering the smart ‘nanotechnological’ products offered by nature, namely, a multitude of living beings characterised by hierarchic structures, evolved and optimised over million years. Such unique structures are capable to provide outstanding performance that has always captured the attention of mankind throughout the ages. Today, copying the unique properties of natural organisms is becoming the target of material scientists seeking a new generation of smart devices. Focusing on bone regeneration, it was found that natural vegetal species such as some woods have a structural organisation and morphology strongly resembling human bones [168]. Woods are indeed cellular materials characterised by a hierarchical porous structure enabling effective vascularisation, even along tens of metres, and also yielding an exceptional combination of high stiffness, toughness, and strength at low density.

In order to use woods as models to be copied and transformed into new inorganic functional devices, new methods based on biomorphic transformation processes has been explored for some years [169,170,171,172]. This approach pursued the complete chemical conversion of woods by maintaining the original multiscale structure. To be performed effectively, the process requires chemical methods ensuring controlled phase transformation, whereas also controlling the structural variations related to such a phase change in order to retain those microstructural features relevant for osteoconduction, vascularisation, and mechanical performance. Among the relatively few approaches attempted so far, poorly effective in respect to maintenance of bioactive composition and relevant mechanical properties, the use of a sequence of heterogeneous chemical reactions occurring between reactive gases and a solid template was recently proposed [173]. This approach permitted researchers to chemically convert a natural rattan wood to a final apatitic scaffold retaining bioactive composition, nanostructure, and the original 3D multiscale structure of the wood. The rattan wood was selected as a bone model for its outstanding similarity with the structure of osteons, which constitute a main functional unit of the long bone hierarchical architecture. The kinetic control of the various reaction consented to use lower process temperatures, strongly limiting the grain growth.

**Figure 5:**
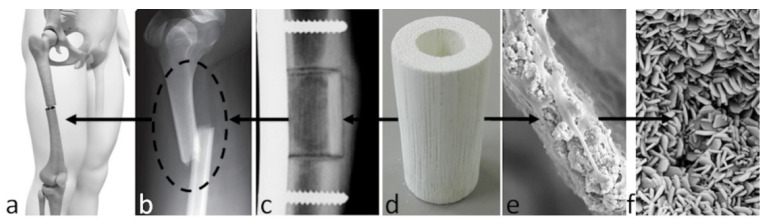
Biomorphic scaffolds for long bone regeneration and their application in long bone defects: (**a**) example of implantation site; (**b**) critical size defect; (**c**) radiography of implanted scaffold; (**d**) bone scaffold from wood; (**e**) scaffold–cell interaction, 250×; (**f**) scaffold nanostructure, 25,000×.

Such a biomorphic transformation process was developed as a heterogeneous gas–solid reaction. After the pyrolysis of the wood, aimed at eliminating all the organic components and obtaining a pure biomorphic carbon template, calcium, phosphate, and carbonate ions were progressively introduced, thus converting the original wood into calcium carbide, calcium oxide, and calcium carbonate phases, followed by a hydrothermal treatment converting the calcium carbonate into the final calcium phosphatic composition. The scaffolds generated by this method are characterised by a unique 3D hierarchic structure on a cellular micro and nanostructure scale, showing a lacunar fractal porosity, inherited from the original wood used as a template, that yielded damage-tolerant mechanical performance close to that of bone [174,175] (Figure 5).

This feature is thus very promising to activate mechanotransduction phenomena at the cell level, which is considered as a major mechanism providing the bone with the ability to respond to complex mechanical forces, as well as to self-repair and self-regenerate upon damage of limited entity [176,177]. Although these biomorphic scaffolds are pure ceramic materials, their unique performance is very different from those of a conventional sintered ceramic body, characterised by brittle fracture mechanisms [174]. Therefore, these achievements promise to pave the way to new bone scaffolds with effective regenerative ability when applied to large, load-bearing bone regions [168].

Relevant aspects of such a biomorphic transformation process were the use of reactive gases in supercritical conditions and high pressure, as well as of wet conditions permitting to better regulate the kinetics of the various chemical reactions yielding the phase transformation in order to achieve highly reactive intermediate precursors facilitating further reactions. More specifically, such a high degree of control was relevant to ensure, at every step of the process, (i) the formation of the desired inorganic phase, (ii) the prevalence of nucleation processes rather than growth, thus helping to maintain high reactivity enabling the subsequent transformation step, and (iii) the maintenance of the biomorphic multiscale hierarchic structure. The *ensemble* of these features was key in reaching outstanding biologic ability, detected in a bioreactor study with stem cells, and showing overexpression of various genes involved in bone regeneration, compared to a sintered apatitic scaffold. In a different study, it was found that the unique 3D hierarchic structure, resembling the vascular network of trees, could favour the crosstalk between stem and endothelial cells and therefore is promising for the effective development of vascular vessels in vivo [175]. In this respect, a recent animal study showed that a biomorphic apatitic scaffold could induce the formation of new, structurally organised bone, even when implanted in an ectopic site. This result is of great relevance because it demonstrates that autologous stem cells can be guided and instructed to form tissues with functional architecture simply by chemical and topological information inherent in the scaffold composition and structure, without using any other osteoinductive or growth factors. These results, which indicate a combination of outstanding in vitro and in vivo osteoinductive ability and bone-mimicking mechanical performance, are very promising for future application of biomorphic scaffolds in more relevant preclinical and clinical tests for regeneration of long, load-bearing bone segments.

## 6. Conclusions and Future Perspectives

The pressing need for scaffolds with effective regenerative abilities, particularly referred to as musculoskeletal tissues, is urging material scientists to find radically new approaches to develop devices with high mimicry of host tissues. This feature is increasingly recognised as a key factor to achieve cell-instructive ability and minimise adverse foreign body reactions so as to promote and sustain the natural metabolic processes yielding tissue regeneration. This task is made very difficult by the intricate combination of composition, texturing, and 3D porous architecture of bone and osteocartilaginous tissues, quite problematic to mimic and to translate, as a whole, in an inorganic implantable device, due to insurmountable technological barriers related to the classical ceramic technology. Notwithstanding, chemically driven assembling processes can permit the consolidation of bone cements to be used as ceramic scaffolds without employing high-temperature treatments, detrimental for the scaffold bioactivity. Looking to natural phenomena and organisms as models to be reproduced, pH-mediated biomineralisation processes can generate highly bioactive nanostructured scaffolds with high regenerative ability in various bone and osteocartilaginous districts. On the other hand, in response to the need to achieve relevant mechanical performance and conducive porosity at the same time, biomorphic transformation processes can be considered a new concept in material science, uniquely able to give 3D hierarchically organised bioceramic scaffolds exhibiting great osteoinductivity and bone-mimicking mechanical performance, thus encouraging further investigations and clinical development.

To summarise, 3D materials with superior performances, obtained by nature-inspired approaches, can be prefigured as a future generation of smart advanced materials. In particular, biomorphic transformation processes potentially offer valuable and flexible tools for scientists also outside the biomedical area, i.e., in all application fields where the combination of chemical composition, structural hierarchy, and mechanical performance is functionally relevant, such as in energy, optics, photonics, mechanics, and metamaterials. Indeed, the great variety of natural structures characterised by outstanding mechanical performance related to their unique 3D architecture represents living models that in the incoming decades can inspire a new generation of smart devices capable of unpreceded applications.

## Figures and Tables

**Table 2:** Methods of synthesising hydroxyapatite nanoparticles.

Method	Synthesis Conditions	Characteristic of HA
Precipitation	- Precipitation - Simple setup - Temperature: RT −80 °C	Preferentially rod-like morphology High production of pure product
Hydrothermal	- Dissolution–precipitation - Inside a reactor - Temperature: 100–250 °C - Pressure: 1–50 atm	Versatility of morphology (from rod- to plat-like)
Sol–gel	- Hydrolysis–condensation - Temperature: RT −80 °C	Finest HA nanoparticles (up to 20–50 nm)
Biomimetic deposition	- Nucleation–growth (via SBF) - Temperature: 37 °C	Applied to make bone-like nanocrystals apatite layer
Microwave	- Dissolution–precipitation - Inside a microwave oven - Temperature: 100–250 °C - Pressure: 1–50 atm	Smaller particle size Good purity Closer size distribution

**Table 3:** Main calcium phosphates involved in CPC products.

Acronym	Compound	Chemical Formula	Ca/P Molar Ratio	Solubility (pK_s_)	pH Stability
MCPM	Monocalcium phosphate monohydrate	Ca(H_2_PO_4_)_2_·H_2_O	0.5	1.14	0.0–2.0
DCPD	Dicalcium Phosphate Dihydrate (Brushite)	CaHPO_4_·2H_2_O	1.0	6.6	2.0–6.0
α-TCP	α-Tricalcium Phosphate	α-Ca_3_(PO_4_)_2_	1.5	25.5	does not precipitate in aqueous solution
β-TCP	β-Tricalcium Phosphate	β-Ca_3_(PO_4_)_2_	1.5	29.5	does not precipitate in aqueous solution
CDHA	Calcium Deficient Hydroxyapatite	Ca_10−x_(HPO_4_)_x_(PO_4_)_6−x_ (OH)_2−x_ (0 < x < 1)	1.5–1.67	<42.6	6.5–9.5
HA	Hydroxyapatite	Ca_10_(PO_4_)_6_(OH)_2_	1.67	58.6	4.5–12.0
TTCP	Tetracalcium phosphate	Ca_4_(PO_4_)_2_O	2.0	37–42	does not precipitate in aqueous solution

**Table 4:** List of relevant CPCs and characteristics of their synthesis.

	Reactives	Product	Type of Reaction	Setting Mechanism
Apatitic	α-TCP (single component)	CDHA	Hydrolysis	α-TCP (dissolution) → CDHA (precipitation)
Apatitic	TTCP + DCPD (multiple component)	HA	Acid-Base	TTCP/DCPD (dissolution) → HA (precipitation)
Brushitic	β-TCP + MCPM (multiple component)	DCPD	Acid-base	β-TCP/MCPM (dissolution) → DCPD (precipitation)

**Table 5:** Example of polymeric additives for alternative formulations of CPCs.

Additives	CPCs Findings	References
Alginate	improved the injectability, high porosity, stronger and easy to handle	[77,78,79]
Chitosan	improved cohesion, higher compressive strength, prolonged setting time	[78,80]
Collagen	increase of new bone formation, increase in resorption rate	[81,82]
Gelatine	porogen, prolonged final setting time, promote cell adhesion, enhanced degradation, improve handling and cohesion	[83,84]
Hyaluronic acid	facilitates bone repair effects by accelerating osteogenic expression, more bone formation, higher osteogenic promoting factors secretion	[85,86]
Hydroxypropyl methylcellulose	good injectability and cohesion, reduced the setting time, increased the porosity after hardening, especially the macroporosity, improved the mechanical properties strong toughening and strengthening effect)	[87,88]
PLGA microspheres	porogen, accelerate the degradation, increase plasticity	[89,90]
Starch	porogen, increasing of setting time, detrimental effect on the compressive strength	[91,92]

**Table 6:** Characteristics of most useful natural polymers (protein- or polysaccharide based) used to fabricate 3D hybrid scaffolds.

	Characteristics	Advantages	Disadvantages	References
Collagen	- Animal-derived fibrous glycoproteins - Most abundant protein in the human body - Fibrous morphology - Primary component in bone - Collagen type I is prevalent in skin, tendon, and bone, while type II is prevalent in cartilage	- Biocompatible - Biodegradable - Mimic native bone - Rich in surface-binding sites for cells - Stimulates - cell adhesion, proliferation, and differentiation	- Low stability/degradation - Difficult processing - Viral and prion contamination - Poor mechanical properties	[120,124,141,142]
Gelatin	- Derives from hydrolysis of collagen - Producing hydrogels with excellent thermostability	- Biocompatible - Biodegradable - Good cell recognition - Easy to mould - Low antigenicity - Good adhesion, proliferation, and differentiation of cells	- Chemical cross-linking needed - Poor mechanical properties - Low stability	[143,144,145,146]
Silk fibre	- Protein fibre secreted by arthropods - Consists of two main proteins, sericin, and fibroin	- Biocompatible - Biodegradable - Slow degradation - High mechanical properties - High thermal stability - High mechanical strength	- Low availability - Residues of contaminants - Brittleness	[147,148,149]
Chitosan	- Natural polysaccharide deriving from chitin (a component of crustacean exoskeleton) - Cationic nature - Relevant - vector for non-viral gene delivery	- Biocompatible - Biodegradable - Biologically renewable - Antibacterial properties - Highly availability - Fast degradation	- Low mechanical strength - Long delay in bone formation - Weak stability - Immunogenicity	[150,151,152]
Alginate	- Natural polysaccharide deriving from brown seaweed or produced by some bacteria - Most abundant marine biopolymers	- Biocompatible - Biodegradable - Easy functionalisation - Resistance to acid conditions - Negatively charged - Form soft hydrogels	- Low mechanical properties - Uncontrolled degradation - Difficult of handling	[153,154,155]
Cellulose	- Natural polysaccharide consisting of a linear chain - Structural component of the primary cell wall of green plants, many forms of algae, and oomycetes	- Biocompatibility - Bioactivity - Biomechanics - Easily converted into derivatives	- Low degradability - High retraction of cellulose hydrogels upon dehydration	[136,145]

## Data Availability

Not applicable.
